# Scoring Cercospora Leaf Spot on Sugar Beet: Comparison of UGV and UAV Phenotyping Systems

**DOI:** 10.34133/2020/9452123

**Published:** 2020-08-05

**Authors:** S. Jay, A. Comar, R. Benicio, J. Beauvois, D. Dutartre, G. Daubige, W. Li, J. Labrosse, S. Thomas, N. Henry, M. Weiss, F. Baret

**Affiliations:** ^1^INRAE, UMR 114 EMMAH, UMT CAPTE, F-84914 Avignon, France; ^2^HIPHEN SAS, 84000 Avignon, France; ^3^ARVALIS-Institut du végétal, 84000 Avignon, France; ^4^Florimond Desprez, 59242 Capelle-en-Pévèle, France

## Abstract

Selection of sugar beet (Beta vulgaris L.) cultivars that are resistant to Cercospora Leaf Spot (CLS) disease is critical to increase yield. Such selection requires an automatic, fast, and objective method to assess CLS severity on thousands of cultivars in the field. For this purpose, we compare the use of submillimeter scale RGB imagery acquired from an Unmanned Ground Vehicle (UGV) under active illumination and centimeter scale multispectral imagery acquired from an Unmanned Aerial Vehicle (UAV) under passive illumination. Several variables are extracted from the images (spot density and spot size for UGV, green fraction for UGV and UAV) and related to visual scores assessed by an expert. Results show that spot density and green fraction are critical variables to assess low and high CLS severities, respectively, which emphasizes the importance of having submillimeter images to early detect CLS in field conditions. Genotype sensitivity to CLS can then be accurately retrieved based on time integrals of UGV- and UAV-derived scores. While UGV shows the best estimation performance, UAV can show accurate estimates of cultivar sensitivity if the data are properly acquired. Advantages and limitations of UGV, UAV, and visual scoring methods are finally discussed in the perspective of high-throughput phenotyping.

## 1. Introduction

Cercospora Leaf Spot (CLS) caused by Cercospora beticola is one of the most damaging foliar diseases for sugar beet (Beta vulgaris L.) crops. It can induce losses of 30 to 48% in recoverable sucrose as reported by [1]. CLS is a polycyclic disease whose severity depends on weather conditions [2]. In warm, wet, and humid conditions, fungus conidia infect leaves, resulting in the appearance of millimeter-scale brown round spots. These necrotic spots then expand and coalesce, eventually defoliating the entire plant and requiring it to grow new leaves. Fungicide treatment may be effective in controlling the development of CLS. However, a significant reduction of the use of fungicides is highly desired since they affect the environment while being expensive [3]. Moreover, their efficacy has already decreased as resistance to fungicides has been reported [4–6]. In addition to crop rotation, such reduction may be achieved with the selection of resistant cultivars and with an early detection of the symptoms enabling a more effective use of fungicides.

For cultivar selection and precision agriculture, CLS symptoms are usually evaluated visually by experts, e.g., based on a scoring scale ranging from 1 for a healthy canopy to 9 for a fully necrosed canopy (Table 1). Visual assessment is often considered as the standard method due to its good accuracy, its ease of implementation, and generally, the lack of available alternatives. However, visual assessment may show some slight variability among experts and times of scoring due to the part of subjectivity in the measurement [7]. An appropriate disease assessment method should indeed be accurate, precise, and reproducible [8]. Several alternative assessment methods have been shown to be more accurate and precise than visual assessments, including counting the number of abscessed leaves in peanuts [9]. Unfortunately, they are still labor intensive and far from being high throughput as required for routine CLS scoring.

Alternatively, several sensor measurements can supplement visual scoring to assess disease symptoms. For example, spectrally based assessment has received increased attention since the first review of Nilsson [10]. Effective use of reflectance measurements for disease detection relies on the identification of relevant spectral features that are, ideally, specific to the targeted disease [11–17]. In the case of CLS, most symptoms correspond to necrotic spots characterized by the loss of green chlorophyll pigments and the synthesis of polyphenols responsible for the brownish color of spots. Such symptoms could be successfully detected using the Cercospora Leaf Spot Index (CLSI) [15] that accurately discriminates CLS infected leaves from healthy, sugar beet rust, and powdery mildew-infected leaves at the leaf scale. At the canopy scale, standard vegetation indices such as the Normalized Difference Vegetation Index (NDVI) [18] were also shown to be accurate indicators of CLS severity [7, 19], as CLS basically reduces the green fraction (GF). However, it remains difficult to discriminate defoliation due to CLS and defoliation due to other sources (natural senescence, diseases, or pests) based on the spectral signature alone, especially under field conditions. Image-based assessment of disease symptoms represents an interesting alternative to spectrally based assessment [20, 21]. Visual analysis of images of individual leaves was first proposed by [22] since computer-assisted image processing was not very mature at that time. Later, some authors proposed to apply image analysis to whole plots [23]. The use of RGB images makes it possible not only to identify the necrotic spots based on their colors but also to characterize their sizes, shapes, and numbers if the spatial resolution is sufficiently fine, which may provide critical information on the disease stage [24].

To carry these sensors, vectors such as UAVs (Unmanned Aerial Vehicles) and UGVs (Unmanned Ground Vehicles) are now capable to reach the high throughput required by the breeders. Both vectors offer specific advantages and drawbacks: UAVs have a very high throughput at relatively low cost [25] at the expense of a sensitivity to illumination and wind conditions. Conversely, UGV can carry active sensors that make the measurements fully independent from the illumination conditions at the expense of a lower throughput and sensitivity to soil conditions. In addition, UGV can easily provide the submillimeter resolution required to identify the CLS symptoms at the earliest stages because of the short distance between crops and sensors. Conversely, although UAVs can reach such high spatial resolution [26], the flight control and data preprocessing are more complex.

The objective of this study is to compare the use of centimeter resolution multispectral imagery acquired from a UAV under passive illumination conditions and submillimeter resolution RGB imagery acquired from a UGV under active illumination conditions, for scoring CLS symptoms in sugar beet phenotyping field experiments. In the following section, the experiments, data collection, and estimation methods are described. The results are then presented in the third section and discussed in the fourth section, with due attention to the advantages and limitations of UGV and UAV systems as compared to the reference visual scoring method.

## 2. Materials and Methods

### 2.1. Field Experiments

**Figure 1:**
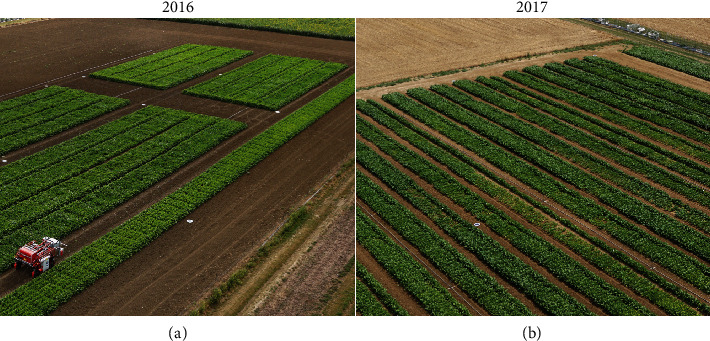
Microplot experiments as observed from the UAV and conducted in 2016 (a) and 2017 (b). In 2016, only the 80 microplots seen in the top right corner of the image were used to study cultivar resistance to CLS. In 2017, all the microplots present in the image were used.

Two microplot experiments were conducted in 2016 and 2017 in Casteljaloux, France (44°19′04N 0°07′E), as illustrated in Figure 1. These experiments were designed to provide a wide range of CLS symptoms, ranging from healthy canopies to fully necrosed canopies. Each microplot had 4 rows of 1.80 m length and spaced by 0.50 m, with a plant density of 11.1 plants/m^2^. In 2016, 80 microplots were monitored, corresponding to 20 genotypes and three treatments. For the first treatment, plants were inoculated with the Cercospora beticola fungus on 07/06 and no fungicide was applied afterward. This treatment was replicated twice. For the second treatment, plants were not inoculated, and no fungicide was applied to represent natural infection by CLS. For the third treatment, plants were not inoculated and fungicide was applied. Each treatment was organized in a line with a random location of the genotypes. In 2017, 1374 genotypes corresponding to the whole reference panel of a breeder were inoculated on 07/11 and no fungicide was applied afterward. Based on previous experiments, the CLS sensitivity was available for 143 of these genotypes. These 143 genotypes could be classified into four classes corresponding to very resistant (15 genotypes), resistant (41 genotypes), sensitive (68 genotypes), and very sensitive (19 genotypes).

### 2.2. Visual Scoring of CLS Symptoms

**Figure 2:**
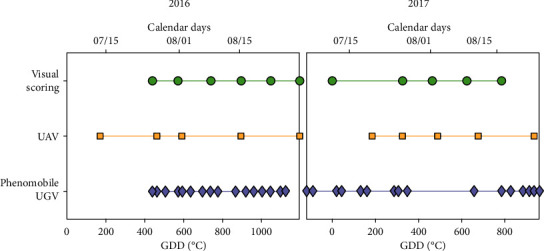
Sampling dates for visual scoring (green circles), UAV (orange squares), and Phenomobile UGV (purple diamonds) measurements, for 2016 (left) and 2017 (right). Time is expressed both in growing degree days (GDD) after disease inoculation (bottom *x*-axis) and in calendar days (top *x*-axis).

For both years, the same expert visually scored CLS severity based on a scale ranging from 1 to 9 (Table 1) and designed by the breeder Florimond Desprez (Florimond Desprez, internal communication). In 2016, the microplots were scored six times (Figure 2) with noninteger values obtained by averaging the two integer score values assigned to the two half microplots, respectively. In 2017, the microplots were scored five times (Figure 2) with a single integer value given by the expert.

### 2.3. Phenomobile UGV RGB Measurements

#### 2.3.1. Data Acquisition

**Figure 3:**
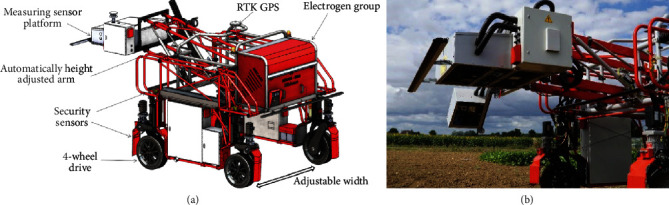
The Phenomobile system: schematic diagram (a) and measurement head (b).

The Phenomobile [27] was a high clearance (1.30 m) UGV with four-wheel drive and steering (Figure 3). It weighted about 900 kg and could reach up to 0.5 m/s speed. The axle track could be changed from 1.50 m to 2.10 m. An arm placed at the front of the vehicle carried the measurement head (Figure 3). Thanks to a Sick LMS 400 lidar, the height of the measurement head was automatically adjusted over each microplot to keep a constant distance of 1.50 m from the top of the canopy. The system was powered by electricity produced by a thermic engine that provided approximately eight hours of battery life. The Phenomobile followed a trajectory that had been initially measured at sowing with an Ashtech MB800 (Trimble® Integrated Technologies, CA, USA) RTK GPS based on a Virtual Reference Station (VRS) network, allowing centimeter positioning accuracy. A similar RTK GPS system was also used by the Phenomobile to ensure centimeter positioning accuracy. The Phenomobile moved automatically over the microplots according to this trajectory and stopped if the VRS connection was lost. When the Phenomobile entered a microplot, several sensor measurements were performed according to a predefined scenario.

To detect CLS spots, one RGB camera pointing nadir was embedded on the measurement head. Four Phoxene FR60 Xenon flashes (http://www.phoxene.com/) with a tunable energy level ranging from 5 to 100 J were synchronized with the RGB camera to make the measurements fully independent from the illumination conditions. Identification of CLS symptoms required a very high spatial resolution that was provided by the Baumer HXG-40 RGB camera (http://www.baumer.com/). This 2048 × 2048 pixel camera was equipped with a 25 mm focal length lens providing a 0.36 × 0.36 mm pixel size at 1.5 m distance with a 0.74 × 0.74 m footprint. RGB images were encoded in 12 bits and saved as 16-bit images in TIFF format.

For each microplot, the Phenomobile passed over each of the four rows at 0.1 m/s speed. It acquired only one image per row in 2016, and two images per row in 2017. The RTK GPS ensured that each image was taken exactly at the same location across the several sampling dates, thus providing a very high spatial consistency. In 2016, the 80 microplots were sampled in less than one hour and at 16 dates (Figure 2). In 2017, the 1374 microplots were sampled in five hours. However, only a subsample of all microplots was sampled at each of the 16 dates (Figure 2), resulting in five to nine observation dates for each microplot.

#### 2.3.2. Variable Extraction from RGB Images

**Figure 4:**
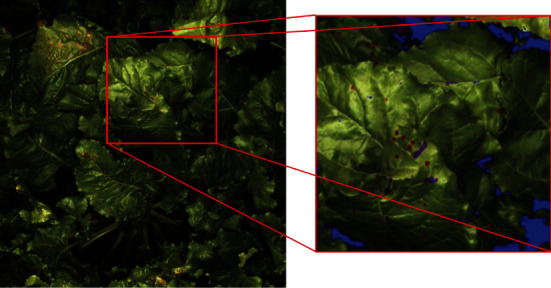
On the left, original RGB image acquired with the Phenomobile using the four flashes. On the right, results of image processing after SVM classification and morphological operations on the areas delimited in red on the original image. Blue pixels are invalid pixels, purple pixels are pixels labeled as nongreen but not identified as CLS spots, and red pixels are pixels labeled as nongreen and identified as CLS spots.

First, saturating pixels that were defined as pixels with red, green, and/or blue band value(s) equal to 2^12^-1 were considered as invalid and removed from the analysis (Figure 4). They were mainly corresponding to strong specular reflections caused by the waxy surface of leaves and stalks. Underexposed pixels that were defined as pixels with luma values lower than 1% of the maximum value were also discarded, which allowed us to remove shaded pixels. On average, saturating and underexposed pixels corresponded to 16% of the whole image, this fraction ranging from 0 to 60% in a few extreme cases (Table 2). Remaining green and nongreen pixels were then classified using support vector machine (SVM) [28, 29] implemented within the Matlab 9.5.0 function “fitcsvm,” as SVM is one of the most powerful classification methods [30]. To create the training and validation datasets, we selected 100 images with maximum variability, i.e., corresponding to several acquisition dates and both years, several microplots, and strong differences in the illumination conditions. For each image, 30 pixels were randomly drawn and assigned to the class “green” or “nongreen,” resulting in a total dataset of 3000 samples. An SVM model with Gaussian kernel and taking as inputs the three RGB bands was trained using 70% of the total dataset. When validated on the remaining 30%, the model showed a 98% classification overall accuracy, defined as the number of correctly classified samples divided by the total number of samples (see the confusion matrix in Table S1 in the supplementary data). To remove isolated green and nongreen pixels due to the classification noise, we applied the same morphological operation to the SVM output binary mask and its logical negative, i.e., an opening by reconstruction based on a disk-shaped structuring element with a radius of 3 pixels (Matlab 9.5.0 function “imreconstruct”). The green fraction (GF), defined as the fraction of green pixels with respect to the total number of valid pixels, was then computed for each image (Table 2). CLS spots were identified based on their shapes and sizes using the Matlab 9.5.0 function “regionprops.” For each group of connected nongreen pixels, the area and eccentricity features were computed to identify CLS spots that were defined as disk-shaped objects with a diameter lower than 4 mm and eccentricity lower than 0.9. This allowed us to effectively discard most of the nongreen pixels corresponding to soil background, necrotic leaf tissues, and remaining stalks. For each image, the spot density (SD), defined as the number of CLS spots divided by the area of valid pixels, and the average spot size (SS), defined as the average diameter of spots in the image, were computed (Table 2). The GF, SD, and SS values were finally averaged over all the images available for each date and each microplot.

### 2.4. UAV Multispectral Measurements

#### 2.4.1. Data Acquisition

An AIRPHEN multispectral camera (http://www.hiphen-plant.com/) was embedded on a hexacopter and fixed on a two-axis gimbal. The camera was equipped with an 8 mm focal length lens and acquired 1280 × 960 pixel images using a 3.6 × 4.8mm CCD sensor. These images were saved in TIFF format at a 1 Hz frequency. The AIRPHEN camera is made of six individual cameras spaced by a few centimeters and sampling the reflected radiation at bands centered on 450, 530, 560 (in 2017, the 570 nm band replaced the 560 nm one), 675, 730 and 850 nm, with a spectral resolution of 10 nm. For each individual camera, the integration time was adjusted automatically to minimize saturation and maximize the dynamics.

The flight plan was designed to ensure 80% overlap across and along track. The UAV was flown at 20 m in 2016 and 50 m in 2017, corresponding to spatial resolutions of 0.9 and 2.3 cm, respectively. Several circular panels of 60 cm diameter were placed evenly within the field and used as ground control points (GCPs) for photogrammetric processing (Section 2.4.2). Their positions were measured using an RTK GPS providing an accuracy of 2 cm. Further, a 3 m^2^ radiometric gray reference panel was used for radiometric calibration [31]. Illumination conditions were generally stable during the flight, except at the fourth date in 2016 (Figure 2) due to intermediate cloud cover and wind. For each year, the UAV was flown five times after CLS inoculation (Figure 2).

#### 2.4.2. Data Preprocessing and Variable Extraction from Multispectral Images

As the six bands were acquired from different points of view, they were first registered using the algorithm proposed by [32] and already successfully used in [31]. This algorithm is based on spatial frequency analysis through the Fourier-Mellin transform, which allows it to solve the visible-near-infrared band registration problem observed with classical scale-invariant feature transform descriptors [33]. A unique band (530 nm) could then be used within the photogrammetric software Agisoft Photoscan Professional edition (Version 1.2.2, Agisoft LLC., Russia) to estimate the camera position for each image acquisition using the GCPs placed in the field. Images could then be projected onto the ground surface with an accuracy of 2 cm when evaluated over the GCPs (1 cm along the *x*- and *y*-axes, and 1.7 cm along the *z*-axis), which was sufficient with respect to the microplot dimensions of 2 × 1.80 m (Section 2.1). Microplots that were fully covered by images acquired with viewing zenith angles lower than 10° (to limit bidirectional effects) were finally extracted. To remove the influence of spectral variations in the incoming light, the digital number, DN^*i*^(*x*, *y*), of each pixel (*x*, *y*) and each band *i* was converted to bidirectional reflectance factor, BRF^*i*^(*x*, *y*) [34], according to the following:

(1) $$\begin{array}{c} {\text{BRF}}^{i}\left(x,y\right)=\left(\frac{{\text{DN}}^{i}\left(x,y\right).v^{i}\left(x,y\right)}{t^{i}}\right).\left(\frac{t_{\text{ref}}^{i}}{\bar{{\text{DN}}_{\text{ref}}^{i}v^{i}}}\right).{\text{BRF}}_{\text{ref}}^{i}, \end{array}$$

where *t*^*i*^ and *t*_ref_^*i*^ are, respectively, the integration times of the images acquired over the microplot and the reference panel, *v*^*i*^ is the vignetting correction factor [31], and $\bar{{\text{DN}}_{\text{ref}}^{i}v^{i}}$ is the pixel-averaged and vignetting-corrected DN value observed over the reference panel of known BRF value, BRF_ref_^*i*^.

Due to the too low spatial resolution, CLS spots could not be identified individually. CLS symptoms were thus assessed using the GF computed by thresholding the Visible Atmospherically Resistant Index (VARI) image [35], as further detailed in [31]. This whole multispectral image processing chain, ranging from the registration of multispectral bands to the estimation of GF, made it possible to estimate GF with a root mean square error of prediction (RMSE) of 0.04, as already validated in [31].

### 2.5. Estimation of Scores Using Phenomobile RGB Data and UAV Multispectral Data

Because Phenomobile, UAV, and visual measurements were not performed at the same dates (Figure 2), we first interpolated Phenomobile and UAV data to the dates of visual scoring using modified Akima cubic interpolation [36]. A linear regression over the last three points was used to extrapolate 2016 Phenomobile data to the last date of visual scoring. This was justified by (1) the continuous behavior of Phenomobile-measured variables over the entire acquisition period (e.g., Figure S1 in the supplementary data), and (2) the short time interval of three days between the last Phenomobile measurement and the last visual scoring (Figure 2). Similarly, the UAV-derived GF value at the first visual scoring date was computed from the Phenomobile-derived GF value based on a linear model between Phenomobile and UAV GF estimates calibrated on the four remaining dates (RMSE = 0.05, not shown).

The CLS scores were estimated using artificial neural networks. For the Phenomobile, four inputs could be used: GF, SD, SS, and GFn. GFn is a transformation of GF designed to reduce the confounding influence of crop growth and CLS development: GF is first divided by the maximum GF value in the time series, and values observed before this maximum are set to 1.0. For UAV data, only GF and GFn could be used as inputs to the neural network. A simple architecture based on a layer of four tangent-sigmoid transfer function neurons followed by a single linear transfer function neuron was used (see Figure S2 in the supplementary data). The default implementation of the Matlab 9.5.0 function “train” was used to train the neural network, i.e., input(s) and output were first mapped to [-1; 1], and the mean square error was optimized using the Levenberg-Marquardt algorithm [37, 38] to estimate weights and biases. Predicted scores lower than 1 or greater than 9 were set to 1 or 9, respectively.

First, the influence of each input variable on CLS score estimation was investigated by testing every possible combination of input variables, i.e., 15 combinations for Phenomobile data and three combinations for UAV data. For each combination, the RMSE was computed using a twofold cross-validation, i.e., using 2016 data for training and 2017 data for validation, and reciprocally. Due to the strong difference in the number of samples between both years (480 samples for 2016 versus 6870 samples for 2017), we randomly drew 480 samples from the 2017 dataset. This was performed by using a *k*-means algorithm [39] with 480 classes and by randomly drawing one sample per class to ensure a more representative sampling of the dataset. Such random sampling was replicated 20 times to account for sampling variability, and the RMSE was computed over these 20 replicates. The optimal combination of input variables was the one with the lowest RMSE.

Second, based on the optimal combination of input variables, the estimation performance obtained for the total 2016 dataset was evaluated by applying a model trained over the total 2017 dataset. Similarly, the estimation performance obtained for the total 2017 dataset was evaluated by applying a model trained over the total 2016 dataset. In both cases, ten neural networks with similar architecture but initialized with different weight and bias values obtained with the Nguyen-Widrow method [40] were trained. Every score estimate was then given by the median of these ten estimates to reduce estimation uncertainty and to limit the sensitivity of the neural network training convergence to the initial conditions [41–43]. The estimation performance was then evaluated using the absolute and relative (with respect to the mean) RMSE and squared Pearson's correlation coefficient (*r*^2^).

### 2.6. Estimation of Genotypic Sensitivity to CLS

To report differences between microplots in a synthetic way, the integral of CLS scores over time was computed for each microplot. Time was expressed in growing degree days (GDD, in °C) with a base temperature of 0°C rather than in calendar days to better represent sugar beet growth and CLS development and for a better consistency between different years. Such an integral was called Area under the Disease Progression Curve (ADPC) by [44]. It is a synthetic index used to quantify the sensitivity of a genotype to a given disease and is given by

(2) $$\begin{array}{c} \text{ADPC}=\overset{n}{\underset{i=1}{\sum }}\frac{\left(S_{i+1}+S_{i}\right)}{2}\left({\text{GDD}}_{i+1}-{\text{GDD}}_{i}\right), \end{array}$$

where *S*_*i*_ is the CLS score measured at date GDD_*i*_ and *n* is the number of observation dates.

For each microplot, ADPC was computed from Phenomobile- and UAV-derived scores and compared to ADPC computed from visual scores using RMSE and *r*^2^.

For the 143 genotypes of known CLS sensitivity class in 2017 (Section 2.1), visually-, Phenomobile-, and UAV-derived ADPC values were also compared based on their abilities to discriminate these genotypes. For each of the three scoring methods, we first sorted the genotypes in ascending order according to their ADPC values. Then, we computed the abundance of each of the four CLS sensitivity classes by a group of 20 genotypes and assigned it to the average ADPC of the group. The abundance of a class was defined as the percentage of genotypes of this class present within these 20 genotypes. A total of 123 (=143-20) groups of 20 genotypes were selected. A moving window with unity step was used to decrease the influence of possible discontinuities in the abundances related to the sampling of these genotypes.

### 2.7. Repeatability of Visual, Phenomobile, and UAV Measurements

The repeatability of a measurement is usually assessed by comparing the values between several replicates. The repeatability of visual and estimated scores and corresponding ADPC was therefore evaluated based on the two replicates “CLS inoculation and no fungicide” studied in 2016 (Section 2.1). It was quantified by computing the RMSE and *r*^2^ between the scores and ADPC obtained for the two replicates.

## 3. Results

### 3.1. Representativeness of RGB Images Acquired with the Phenomobile

**Figure 5:**
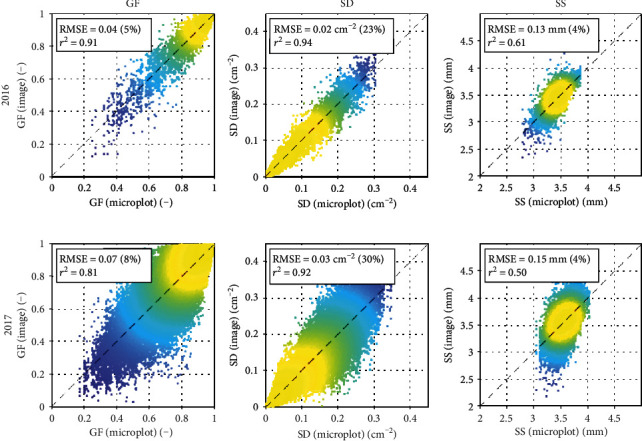
Variability of Phenomobile-derived GF (left), SD (middle), and SS (right) per image (*y*-axis) versus their averages per microplot (*x*-axis). In 2016 (top row), four images per microplot were used and in 2017 (bottom row), eight images per microplot were used. Absolute and relative RMSE and squared Pearson's correlation coefficients are shown. The color indicates the point density, ranging from blue for low density to yellow for high density.

Four to eight RGB images were taken with the Phenomobile for every microplot. We first evaluated the variability between the GF, SD, and SS values derived from single images as compared to their averages per microplot. Results show that such variability remained limited for 2016, with relative RMSE computed over all the microplots and observation dates lower than 23% and *r*^2^ higher than 0.61 (Figure 5). For 2017, the variability was larger for the three variables, as shown by the higher RMSE and lower *r*^2^ obtained as compared to 2016 (Figure 5). Fortunately, the number of images acquired in 2017 (eight) ensured a good representativeness of each microplot.

Such a good representativeness was confirmed by the smooth temporal courses of Phenomobile-derived variables observed for both years before and after interpolation to the dates of visual scoring (Figure S1 in the supplementary data). Note that in the case of UAV, similarly smooth temporal courses were observed for GF and both years (Figure S1 in supplementary data).

### 3.2. Dynamics of Visual Scores and Phenomobile- and UAV-Derived Variables

**Figure 6:**
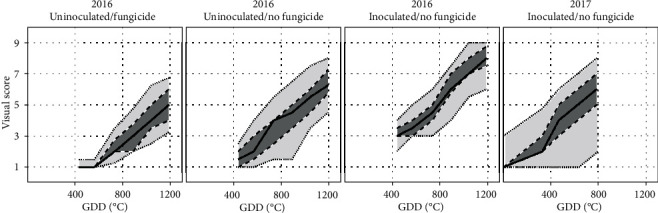
Temporal courses of visual scores for 2016 (first to third plot) and 2017 (fourth plot). For 2016, the three treatments studied are shown: no inoculation and fungicide application (first plot), no inoculation and no fungicide application (second plot), and inoculation and no fungicide application (third plot). For 2017, only one treatment is studied: inoculation and no fungicide application (fourth plot). For each plot, the solid line shows the median value over all microplots, the dark gray area delimits the 25th and 75th percentiles, and the light gray area delimits the 10th and 90th percentiles. Time is expressed in growing degree days (GDD) after disease inoculation.

Regardless of years and treatments, visual scores regularly increased over time (Figure 6). For 2016, uninoculated microplots showed delayed CLS infection when fungicide was applied, the median score reaching only a maximum of five over the studied period. Fungicide was, however, effective only for a limited period, after which natural CLS infection eventually occurred. Without fungicide application, uninoculated microplots generally showed slightly higher scores, indicating that CLS developed faster for this treatment. Inoculated microplots where no fungicide was applied showed an early development of CLS and significantly higher scores than the other two treatments for every date. In this case, all the microplots showed scores higher than five for the last observation date. The microplots conducted similarly in 2017 (inoculation and no fungicide) generally showed lower scores due to the early interruption of measurements (GDD = 785°C) that made it impossible to evaluate the late stages of CLS development for most of the microplots. A significantly stronger variability between microplots was also observed in 2017, probably due to the larger number of cultivars considered and the wider range of sensitivity levels.

Overall, the combinations of several treatments and genotypes in 2016 and several genotypes in 2017 successfully introduced a strong variability in the CLS symptoms, the visual scores generally covering the whole range of possible values (Figure S3 in the supplementary data). The distribution of visual scores was, however, less uniform in 2017, showing a strong proportion of scores of one and very few scores higher than seven (Figure S3 in the supplementary data).

**Figure 7:**
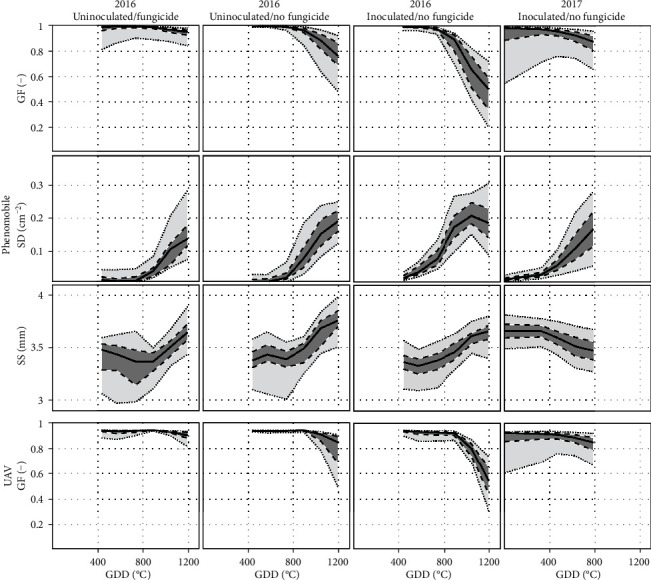
Temporal courses of GF (row 1), SD (row 2), and SS (row 3) derived from Phenomobile RGB imagery, and GF derived from UAV multispectral imagery (row 4), for 2016 (columns 1-3) and 2017 (column 4). For 2016, the three treatments studied are shown: no inoculation and fungicide application (column 1), no inoculation and no fungicide application (column 2), and inoculation and no fungicide application (column 3). For 2017, only one treatment is studied: inoculation and no fungicide application (column 4). For each plot, the solid line shows the median value over all microplots, the dark gray area delimits the 25th and 75th percentiles, and the light gray area delimits the 10th and 90th percentiles. Time is expressed in growing degree days (GDD) after disease inoculation.

Phenomobile-derived variables GF, SD, and SS showed typical temporal profiles associated to the development of CLS symptoms (Figure 7). In 2016, the canopy was nearly fully covering the soil when Phenomobile observations started, with GF ≈ 1.00 (row 1 in Figure 7). GF then decreased regularly over time. The inoculated microplots where no fungicide was applied showed the strongest and earliest decrease (median GF of 0.50 at GDD = 1200°C). On the other hand, the uninoculated microplots where fungicide was applied showed the lowest and latest decrease (median GF of 0.95 at GDD = 1200°C). In 2017, the measurements started at CLS inoculation, when GF was still increasing (row 1 in Figure 7, right plot). Maximum GF was reached at around GDD = 450°C, with GF values significantly lower than 1.00. After GDD = 450°C, GF started decreasing, but measurements ended too early to observe low GF values as seen for the same treatment (i.e., CLS inoculation and no fungicide application) in 2016.

For the three treatments in 2016, the spot density increased over time up to approximately SD = 0.2 cm^−2^ (row 2 in Figure 7). SD increased earlier for the inoculated microplots where no fungicide was applied and then started decreasing. Such decrease was not visible for the other two treatments, probably because measurements ended too early. The same was observed for 2017, i.e., SD increased until the interruption of measurements, the median value reaching SD = 0.17 cm^−2^ at GDD = 785°C.

The average spot size SS showed different temporal profiles for 2016 and 2017 (row 3 in Figure 7). SS slightly increased over time in 2016 (although less strongly for the inoculated microplots), while it slightly decreased in 2017.

UAV-derived GF showed similar temporal courses as Phenomobile-derived GF for both years (row 4 in Figure 7). The main difference between the two vectors was the maximum GF reached, i.e., 0.94 for UAV and 0.99 for the Phenomobile.

### 3.3. Estimation of Visual Scores

**Figure 8:**
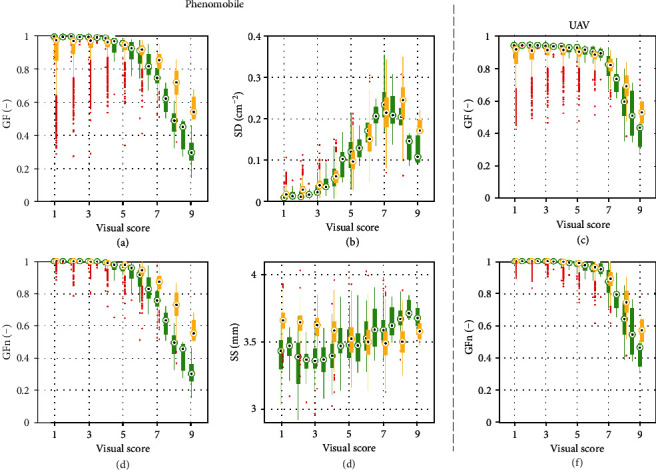
Relationships between visual scores and Phenomobile-derived variables GF (a), GFn (d), SD (b), and SS (e), and between visual scores and UAV-derived variables GF (c) and GFn (f). Green boxes correspond to 2016, and orange boxes correspond to 2017. For each box, the central mark is the median, the edges of the box delimit the 25th and 75th percentiles, the whiskers extend to the most extreme datapoints that are not considered to be outliers, and the outliers are plotted individually in red.

The four features, GF, GFn, SD, and SS, extracted from Phenomobile and/or UAV images generally varied similarly with CLS visual scores for 2016 and 2017 (Figure 8). As expected, Phenomobile- and UAV-derived GF generally decreased as the CLS score increased (Figures 8(a) and 8(c)). For scores greater than five, the decrease was stronger and its rate differed between 2016 and 2017, especially for Phenomobile-derived GF (Figure 8(a)). A large GF variability was also visible for scores lower than five in 2017, while such variability was greatly reduced when considering the normalized GF (GFn). GFn showed a more consistent relationship with the visual score, with limited variations for scores lower than five and strong variations for scores greater than five (Figures 8(d) and 8(f)). The spot density SD was sensitive to variation in the CLS score, even for low scores of 2 or 3. However, the relationship between SD and scores was not monotonic: SD increased with scores up to scores of seven (in 2016) or eight (in 2017) before decreasing. As for its temporal profile, the average spot size SS showed different relationships with CLS visual score for 2016 and 2017. While SS increased with score in 2016, it slightly decreased with score in 2017.

In the case of Phenomobile, the twofold cross-validation process showed that the optimal set of input variables to the neural network for score estimation was GFn, SD, and SS, with RMSE = 0.91 (Table 3). The best explanatory variable was SD, which appeared in the first eight sets of input variables, followed by GFn, which appeared in the best four combinations, while GF and SS only appeared twice. SS only brought marginal information as compared to the use of GFn and SD alone, decreasing the RMSE from 0.98 to 0.91.

In the case of UAV, the best performance was obtained using GFn alone, with RMSE = 1.23 (Table 3). These results were similar to those obtained with Phenomobile-derived GFn (RMSE = 1.19). Conversely, using GF instead of GFn significantly worsened the performance for Phenomobile and UAV: for example, RMSE of 2.30 (for Phenomobile) and 2.35 (for UAV) were obtained using GF alone.

**Figure 9:**
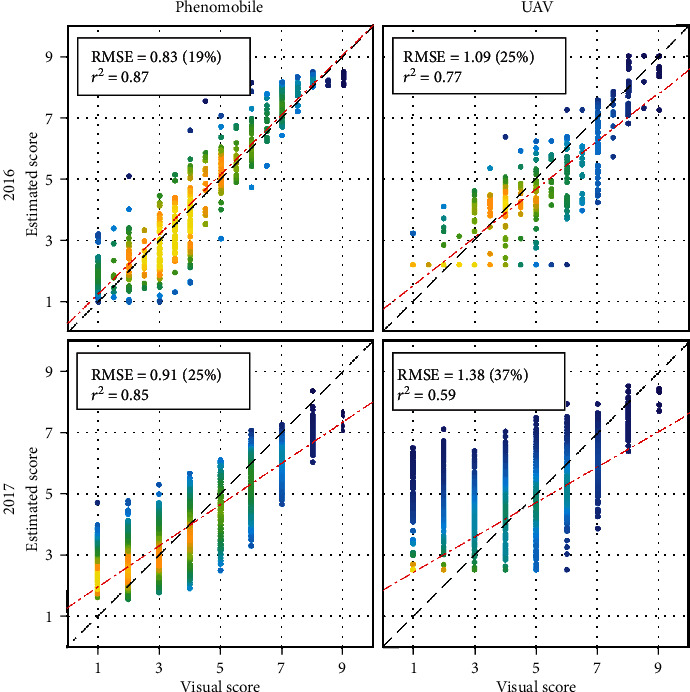
Score estimation results obtained for 2016 (top) and 2017 (bottom) using Phenomobile-derived GFn, SD, and SS (left) or UAV-derived GFn (right) as input(s) to the neural network. For each plot, one year is used for training and the other year is used for validation. Every estimate is the median of ten estimates obtained with ten neural networks. Absolute and relative RMSE, squared Pearson's correlation coefficient, and linear fit are shown. The color indicates the point density, ranging from blue for low density to yellow for high density.

Detailed inspection of the best score estimation results obtained for each year and each vector showed that the performance was consistent across years when using Phenomobile-derived GFn, SD, and SS as inputs to the neural network, with RMSE ≈ 0.87 and *r*^2^ ≈ 0.86 for 2016 and 2017 (Figure 9). However, high scores were slightly overestimated for 2016 and underestimated for 2017. Further, low scores were slightly overestimated for 2017.

Results were less consistent across years when using UAV-derived GFn as input to the neural network (Figure 9): accurate estimates were obtained for 2016 (RMSE = 1.09), while poorer estimates were obtained for 2017 (RMSE = 1.38). High scores tended to be underestimated for 2016, and low scores were generally overestimated for 2017. Further inspection of 2016 results showed that scores were particularly underestimated at the fourth date (Figure 2): a RMSE of 0.87 was obtained when removing this date. For 2016 and 2017, estimated scores showed some saturation for low scores, with a minimum estimated score between two and three. The agreement between estimated and visual scores increased with the score value: for scores greater than seven, the estimation accuracy obtained with the UAV was similar to that obtained with the Phenomobile (Figure 9).

### 3.4. Estimation of Genotype Sensitivity to CLS

**Figure 10:**
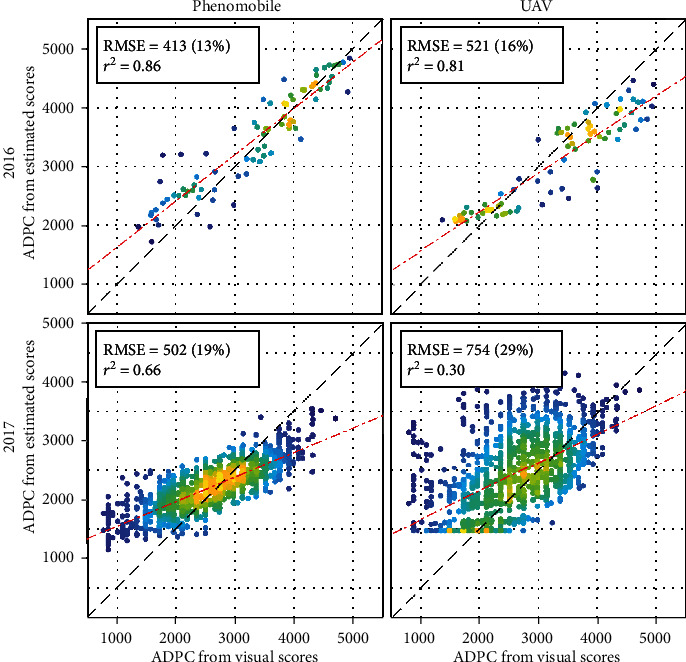
ADPC computed from Phenomobile- (left) and UAV-derived (right) scores (see estimates in Figure 9) versus ADPC computed from visual CLS scores, for 2016 (top) and 2017 (bottom). Absolute and relative RMSE, squared Pearson's correlation coefficient, and linear fit are shown. The color indicates the point density, ranging from blue for low density to yellow for high density.

Phenomobile- and UAV-derived ADPC, that approximate genotype sensitivity to CLS, generally agreed well with ADPC computed from visual scores (Figure 10). Accurate estimates were obtained for 2016, with slightly better results for the Phenomobile (RMSE = 413, ^2^ = 0.86) as compared to the UAV (RMSE = 521, ^2^ = 0.81). In 2017, the Phenomobile provided poorer but still reasonable performance (RMSE = 502, *r*^2^ = 0.66). Conversely, ADPC derived from the UAV showed poor agreement with ADPC derived from visual scores (RMSE = 754, *r*^2^ = 0.30), with a general overestimation and a saturation observed for low ADPC values. Biases were visible on the four plots in Figure 10, generally due to an overestimation of low ADPC values and an underestimation of high ADPC values.

**Figure 11:**
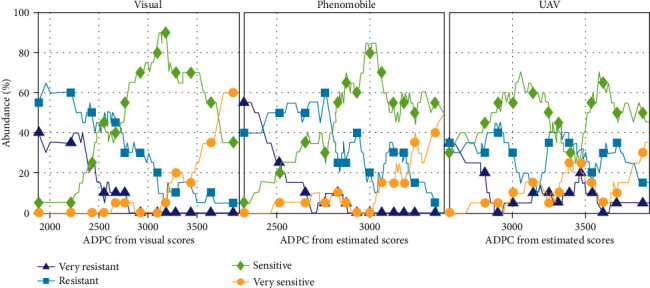
Abundances of the four CLS sensitivity classes (very resistant, resistant, sensitive, and very sensitive) as functions of ADPC computed from visual scores (left), Phenomobile-derived scores (middle), and UAV-derived scores (right) in 2017 (see estimates in Figure 10).

For 2017, ADPC derived from visual scoring and Phenomobile measurements were reasonably consistent with CLS sensitivity classes defined from previous independent experiments (Figure 11): very resistant and resistant classes obtained the lowest ADPC values, while sensitive and very sensitive classes obtained the highest ADPC values. Furthermore, visual scoring made it possible to separate very sensitive and sensitive classes reasonably well; however, it failed to separate resistant and very resistant classes. On the other hand, resistant and very resistant classes were well separated with Phenomobile data; however, the latter failed to separate sensitive and very sensitive classes.

As for UAV measurements, the poor ADPC estimation performance obtained for 2017 (Figure 10) led to poor discrimination of the four classes (Figure 11). In particular, resistant and sensitive classes were inaccurately identified from the ADPC values.

### 3.5. Repeatability of Visual, Phenomobile, and UAV Measurements

**Figure 12:**
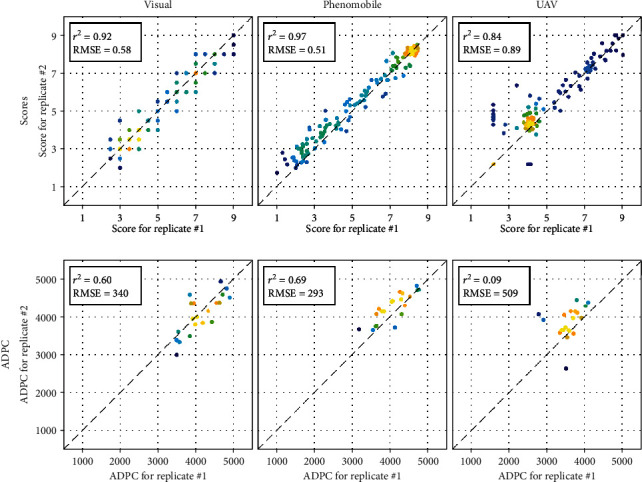
Relationships between the two score (row 1) or ADPC (row 2) values corresponding to the two replicates “inoculation and no fungicide application” in 2016 (see estimates in Figures 9 and 10). Scores and ADPC are derived from visual (left), Phenomobile (middle), and UAV (right) measurements. Absolute and relative RMSE and squared Pearson's correlation coefficient are shown. The color indicates the point density, ranging from blue for low density to yellow for high density.

Among the three scoring methods, Phenomobile RGB imagery provided the most repeatable score and ADPC estimates when evaluated over the two replicates “inoculation and no fungicide application” in 2016 (Figure 12): a strong linear correlation and low RMSE were obtained between replicates, both for score estimates and for corresponding ADPC. Visual scoring showed a slightly lower agreement between replicates (Figure 12). The repeatability of UAV-derived scores was similar to that observed with Phenomobile and visual scores for score values greater than five (Figure 12). Conversely, poor results were observed for lower score values, resulting in lower *r*^2^ (0.84) and higher RMSE (0.89). Consequently, good repeatability was obtained for high ADPC values and low repeatability for low ADPC values. Note that the fourth date (Figure 2) showed poor repeatability as a consequence of the poor score estimates already noticed (Figure 9): when removing this date, RMSE decreased from 0.89 to 0.65 for scores and from 509 to 407 for ADPC.

## 4. Discussion

### 4.1. SD and GFn Are the Best Proxies of CLS Scores

CLS symptoms range from a few brown necrotic spots on some leaves for low severity levels, to a partially or fully necrosed canopy for high severity levels (Table 1). Accordingly, the spot density SD is therefore the best variable to monitor CLS development for scores less than or equal to five (Figure 8). For such low scores, only a minority of millimeter-scale CLS spots join (Table 1), which does not significantly decrease the green fraction GF (Figure 8). In this case, scores and GF can even increase simultaneously as observed for 2017 (Figure 8). Indeed, CLS can infect the crop at different growth stages, including medium development of the canopy for which the increase in GF due to crop growth can be stronger than the decrease in GF due to CLS-induced necrosis. Using the proposed normalized variable GFn instead of the original GF estimate makes it possible to avoid the above confusion since GFn is set to one before the maximum GF is attained. Another advantage of GFn is that the normalization by the maximum GF limits the influence of possible difference in the canopy development as observed between 2016 and 2017 (Figure 8).

For scores greater than five, individual CLS spots join on most of the plants, leading to the necrosis of an increasing number of leaves (Table 1). As a result, GFn, even when derived from UAV centimeter-scale images, becomes the best variable to study CLS development for such advanced disease stages (Figure 8). On the other hand, the nonmonotonic relationship between SD and CLS score makes SD a poor indicator of high scores (Figure 8). Such a behavior is due to (1) the initial spot multiplication that increases SD up to scores of around seven and (2) the coalescence of small individual spots into larger necrosis areas that are no longer identified as spots, which decreases SD for higher scores.

Competition between spot multiplication and spot coalescence may also explain the different temporal courses of the average spot size SS observed for 2016 and 2017 (Figure 7). Appearance of new small spots tends to decrease SS, while merging of older spots into larger ones tends to increase SS. The stronger increase in SS observed for the uninoculated microplots in 2016 as compared to the inoculated microplots in 2016 and 2017 may indicate that spot coalescence prevails over spot multiplication when CLS is not inoculated artificially. In this case, infection may occur in a more localized way, with spots more spatially grouped and thus more chance for them to coalesce. On the other hand, artificially inoculated microplots may show a more homogeneous spot distribution and, therefore, less chance for the spots to coalesce. Anyway, SS does not show consistent relationship with CLS scores over the two years (Figure 8), indicating that this variable brings little information as compared to SD and GFn.

### 4.2. Phenomobile RGB Imagery Provides More Accurate Estimates of CLS Scores than UAV Multispectral Imagery

SD and GFn provide useful information to characterize CLS throughout its development, SD being useful for low scores and GFn for high scores. This explains why SD and GFn appear in each of the best four sets of variables used as inputs to the neural network to estimate CLS scores from Phenomobile RGB imagery (Table 3). The optimal set of variables is GFn, SD, and SS, which indicates that SS contains useful although minor additional information (Table 3). The high accuracy obtained for both years (RMSE ≈ 0.87, *r*^2^ ≈ 0.86) demonstrates the relevance of these three variables and the strong potential of Phenomobile to score CLS symptoms. This is of critical importance as the early detection of disease symptoms in the field is often considered as a major bottleneck for plant breeding and precision agriculture [45].

On the other hand, the poorer performance obtained with UAV multispectral imagery is mainly due to the coarser image spatial resolution that makes it impossible to exploit SD and SS. Using GFn only, UAV multispectral imagery cannot accurately estimate scores lower than five (e.g., see the flat bottoms of the scatter plots on the left-hand side of Figure 9) since these scores correspond to GFn ≈ 1 (Figure 8). This explains the poorer performance obtained for 2017 as compared to 2016, since the 2017 dataset contains a large proportion of low scores and only few scores higher than seven (see Figure S3 in the supplementary data). Note that the difference in the spectral configuration between the two systems only plays a minor role on GF estimation (RMSE = 0.05, see Section 2.5) and therefore on score estimation (see the similar RMSE values obtained with UAV- and Phenomobile-derived GF and/or GFn in Table 3).

Besides coarser spatial resolution, two secondary factors worsen the across-year relationship between UAV-derived GFn and visual scores and contribute to the decrease of the score estimation accuracy for both years: (1) an inaccurate radiometric calibration due to changing illumination conditions during the flight, and (2) the difference in the spatial resolution of the images used in 2016 and 2017. Inaccurate radiometric calibration especially occurred at the fourth UAV acquisition date in 2016 (Section 2.4.1) and caused GF overestimation. Unfortunately, the nearest flights were performed only two weeks after and before the fourth one (Figure 1). Moreover, the fourth date corresponded to the period when GF started decreasing due to CLS development (Figure 7). Therefore, GF was overestimated for the three dates of visual scoring around the fourth UAV acquisition date (Figure 2). This severely worsened the relationship between GFn and visual scores due to the low sensitivity of GFn to score variation for such intermediate scores (Figure 8). The consistency of this relationship across years was also affected by the difference in the spatial resolution between 2016 (0.9 cm) and 2017 (2.3 cm). The finer spatial resolution used in 2016 made GFn decrease for slightly lower scores as compared to 2017, although this was not visible in Figure 8(f) due to the compensation with the GF overestimation caused by inaccurate radiometric calibration. While this shows the interest of increasing the spatial resolution to improve the sensitivity of UAV measurements to score variation, this also emphasizes the need for flying the UAV always at the same altitude.

The score estimation errors presented in Figure 9 are therefore affected by several factors related to the remote-sensing measurement. However, estimation errors are also affected by another nonnegligible factor related to the reference measurement: human errors in the visual scoring leading to a lack of consistency between dates. The lack of consistency is particularly visible when observing the different relationships obtained between GFn (derived from Phenomobile or UAV) and visual scores for 2016 and 2017 (Figure 8): when the score increases, GF shows a stronger decrease in 2016 than in 2017. This explains the apparent overestimation and underestimation of high scores observed in 2016 and 2017, respectively, with the Phenomobile. There were also some errors in the visual scoring for low scores, as demonstrated by a detailed inspection of 2017 Phenomobile RGB images that were showing an overestimation of scores of one and two (Figure 9). A few CLS spots were indeed visible in these images, which means that scores of two and three would have been more appropriate for those microplots (Table 1), as predicted by the neural network. Errors in the visual scoring thus influence the results obtained with Phenomobile and UAV, either in a favorable or in an unfavorable way. This poses the question of using visual scoring for phenotyping purposes as also discussed in Section 4.4.

### 4.3. Cultivar Sensitivity to CLS Can Be Estimated with Both Vectors under Certain Conditions

Cultivar sensitivity to plant disease is often represented with the ADPC synthetic indicator [44, 46]. ADPC is classically given by the integral of visual scores over time. However, such integral can also be computed using scores estimated from Phenomobile or UAV images as done in this paper. The trends observed for scores (Section 4.2) are therefore also found for the corresponding ADPC. In particular, the overestimation of low scores observed with the Phenomobile in 2017 is even more visible on ADPC estimation (Figure 10) due to the high proportion of visual scores lower than or equal to two (e.g., the median visual score did not exceed two during the first half of the campaign, see Figure 6). Still, the accurate score estimates obtained with the Phenomobile result in accurate ADPC estimates for both years (RMSE ≤19%).

Since estimated ADPC is the integral of estimated scores over all the sampling dates, it smoothens out the uncertainties associated with the individual score estimates. Therefore, while the score estimation accuracy obtained with the UAV may not be sufficient for breeders (e.g., RMSE = 25% in 2016), the ADPC estimation accuracy may be acceptable (e.g., RMSE = 16% in 2016). Besides limiting the detrimental influence of external factors that may affect UAV measurements (Section 4.2), performing these measurements sufficiently late in the growing season appears as a simple yet effective solution to improve ADPC estimation from UAV. Indeed, the score estimation accuracy obtained with GFn alone increases with the score value due to the increasing sensitivity of GFn (Figures 8 and 9): for example, the RMSE per score value averaged over 2016 and 2017 is lower than 0.79 for scores greater than or equal to eight, while it is higher than 1.05 for scores lower than eight (data not shown). Computing ADPC based on GFn-derived estimates of scores lower than seven thus provides poor ADPC estimates (Figure 10). This also explains the flat bottom of the scatter plot obtained for UAV estimation in 2017 (Figure 10), which corresponds to the 25% of the microplots whose score values remained lower than five during the studied period (Figure 6). On the other hand, considering scores higher than seven improves ADPC estimation. Such results allow us to define a specific requirement when assessing cultivar sensitivity to CLS from UAV multispectral imagery in phenotyping experiments: UAV flights should be performed until all the microplots reach a maximum score of nine, i.e., when all the microplots are fully necrosed. The objective should be to identify when each microplot reaches this maximum score to get optimal UAV ADPC estimation performance.

While the poor ADPC estimation results obtained in 2017 with UAV prevented an accurate discrimination of CLS sensitivity classes, the classification results obtained with the Phenomobile were promising (Figure 11). Phenomobile could better discriminate the very resistant and resistant classes than visual scoring, which tends to confirm that low scores were not overestimated by Phenomobile but rather underestimated by visual scoring, as suggested in Section 4.2. However, Phenomobile showed poorer discrimination of sensitive and very sensitive classes than visual scoring. This may have been due to the different relationships between GFn and visual scores observed in 2016 and 2017 that caused an underestimation of high scores in 2017 (Section 4.2). Anyway, it is worth mentioning that the classification performance obtained with Phenomobile and UAV measurement would have been probably improved with later measurements.

### 4.4. Phenomobile and UAV Scorings Can Complement Visual Scoring for Sugar Beet High-Throughput Phenotyping

The three scoring methods used in this study (visual scoring, Phenomobile RGB imagery, and UAV multispectral imagery) present advantages and drawbacks that should be properly understood before choosing a scoring method for sugar beet high-throughput phenotyping (Table 4).

#### 4.4.1. Accuracy

Our results suggest that Phenomobile RGB imagery generally provides more accurate score and ADPC estimates than visual scoring. Although based on a unique scoring scale (Table 1), visual measurements remain subjective and prone to errors, as it may be difficult to accurately characterize a gradient in GF for high scores or to detect a few spots in a dense canopy for low scores, based on visual assessment. Our results show that these errors limit the consistency of the across-year relationships between remote-sensing variables and visual scores, which then impacts the performance of machine learning models. Note that this problem would have been even more detrimental if visual scoring had been achieved by different experts. UAV multispectral imagery generally provides poorer score estimates for low to intermediate score values due to its lower spatial resolution. Still, it can provide accurate and consistent estimates of high scores that are similar to those obtained with Phenomobile, thus yielding reasonably accurate estimates of ADPC if UAV measurements are performed until all the microplots have reached a maximum score of nine. However, great attention must be paid to the UAV data acquisition, especially due to the passive nature of the imagery. In particular, images should always have the same spatial resolution and flights should be performed under stable illumination conditions as far as possible to ensure accurate radiometric calibration. If the latter is not possible, computing data quality flags for every microplot and date (e.g., based on the expected reflectance values of vegetation or soil) could allow us to filter out inappropriate data before computing ADPC if UAV measurements have been performed at a sufficiently high frequency, e.g., on a weekly basis.

#### 4.4.2. Specificity

An important weakness of UAV multispectral imagery is its nonspecificity, since the observed variations in GF cannot only be due to CLS but also to weeds, natural senescence, and other diseases such as *Phoma betae*, etc. For that reason, human supervision may be necessary to detect potential problems. Note, however, that this problem is limited in phenotyping experiments because weeds are controlled and CLS is inoculated artificially at the optimal date so CLS can spread before other diseases and natural senescence. The use of SD and SS seemingly makes Phenomobile RGB imagery more specific to CLS; yet, visual assessment by an expert remains the reference method from this point of view.

Representativeness of the measurements is also not an issue for visual scoring, as the expert can visualize the whole microplot before scoring. It is also not an issue for UAV multispectral imagery since only images that fully cover the microplot are used. On the other hand, performing a proper sampling of the microplot with the Phenomobile is trickier because not all the microplot is imaged. Therefore, a sufficiently high number of images should be acquired to capture any possible spatial heterogeneity.

#### 4.4.3. Repeatability

When evaluated over the two replicates in 2016, repeatability of Phenomobile measurements was slightly better than repeatability of visual scoring, even if the discrete nature of visual scores as opposed to the continuous values derived from RGB imagery may explain part of the difference (Figure 12). The greater uncertainty observed for scores lower than five with UAV multispectral imagery explains the poor repeatability observed for those scores. Such scores are poorly sensitive to GFn variation, so their estimation is particularly sensitive to external factors such as inaccurate radiometric calibration in this case. This further emphasizes the need for high scores when performing UAV measurements, as the higher sensitivity of GFn will decrease the influence of such external factors. When evaluated over two years, repeatability of visual scoring is more questionable than that of the other two methods, as discussed above based on the different relationships between GFn and visual scores observed in 2016 and 2017 (Figure 8). Accuracy and repeatability of visual scoring may be particularly affected for very large experiments such as the ones conducted in 2017 because of possible fatigue of the expert induced by the scoring of hundreds or thousands of microplots in a row.

#### 4.4.4. Efficiency

UAV multispectral imagery appears much more efficient than the other two methods, with about 2000 microplots sampled per hour when flying at 50 m. At the same time, only 300 microplots can be sampled with Phenomobile RGB imagery, and 150 (for early CLS stages) to 220 (for late CLS stages) microplots can be sampled with visual scoring. Such a gain turns out to be a critical advantage of UAV multispectral imagery for large phenotyping experiments. It should encourage the respect of the guidelines provided above for this kind of imagery and potentially motivate the development of alternative methods to improve the radiometric calibration of UAV data.

#### 4.4.5. Affordability

In [25], the authors showed that, when including every source of expense (sensor, vector, maintenance, manpower, and training), the UAV system was cheaper than the UGV one by a factor ranging from 1.7 and 3.5. This also turns out to be a critical advantage when choosing one of the two approaches.

## 5. Conclusions and Perspectives

In this study, we assess the use of Phenomobile submillimeter-scale RGB imagery acquired under active illumination and UAV centimeter-scale multispectral imagery acquired under passive illumination, for scoring CLS symptoms in sugar beet phenotyping experiments. These two scoring methods are also compared with the reference visual scoring method. Based on two years and more than one thousand cultivars, the results show that the submillimeter spatial resolution of the RGB imagery and the active illumination conditions provided by the flashes makes the Phenomobile an extremely powerful tool to extract critical features such as SD and GFn, both of which are accurate indicators of low and high scores, respectively. Scores can thus be estimated accurately over their whole range of variation, from healthy green plants to fully necrosed canopies. This is a very important result as the early detection of disease symptoms in field conditions is often considered as a major challenge. Cultivar sensitivity to CLS expressed with the ADPC variable can then be retrieved accurately based on these score estimates. In the case of UAV multispectral imagery, only GFn is available due to the coarser spatial resolution so only high scores can be estimated accurately. Still, UAV multispectral imagery can be used to retrieve ADPC with a reasonable accuracy, provided that (1) measurements are performed sufficiently late in the growing season so that all the microplots have reached the maximum score and (2) the detrimental influence of external factors such as changing illumination conditions leading to inaccurate radiometric calibration is minimized. This would make it possible to take advantage of the stronger efficiency and lower cost of UAV measurements as compared to Phenomobile measurements, without a significant loss in accuracy. The results also show that image-based methods can outperform visual scoring on some aspects, e.g., due to the subjective nature of visual scoring whose effect may be increased when scoring very large phenotyping experiments. This is especially true for very low and very high scores as it may be difficult to see a few brown spots in a dense canopy or to accurately assess a change in GF visually.

Currently, human supervision however remains necessary because of the diversity of biotic and abiotic stresses that can affect sugar beet plants in a similar way. Accurate assessment of cultivar sensitivity to CLS requires the CLS symptoms to be discriminated from those of other diseases such as *Phoma betae* for example. Therefore, Phenomobile or UAV images and corresponding score estimates could be checked visually after image processing to detect a potential problem in the estimation, especially when using UAV-derived scores that are less specific to CLS. This problem could also be solved using more advanced algorithms based on deep learning that would be able to discriminate symptoms [21, 47]. This would require a very large and diversified database to obtain accurate and robust models, the diversity including all the different stresses expected and a wide range of cultivars and canopy structure. However, such database was not available for this study. Finally, an interesting prospect for the UAV would be to estimate GF with classical RGB imagery instead of multispectral imagery. The finer spatial resolution of RGB imagery could indeed lead to a better sensitivity for low scores. The wider spectral bands may slightly decrease the GF estimation accuracy, but this decrease could be compensated for by using more advanced classification algorithms, such as deep learning models or SVM models based not only on RGB features but also on textural features for example.

## Figures and Tables

**Table 1:** Scoring scale used for visual assessment of CLS symptoms (Florimond Desprez, internal communication).

Score	Description
1	No CLS spots.
2	Spots on one leaf or two.
3	Spots multiplication.
4	Spots start to join on one plant or two.
5	Spots join on several plants but not on most of the row.
6	Spots join on most of the plants.
7	Some leaves are fully necrosed. Until 50% of leaf area is destroyed.
8	Just three or four healthy leaves remain on the plants.
9	All the leaves are necrosed.

**Table 2:** Variables extracted from the RGB images acquired with the Phenomobile.

Variable	Definition	Unit	Min	Max	Equation
*P*_*t*_	Total number of pixels per image	—	4.2 × 10^6^	4.2 × 10^6^	—
*P*_*v*_	Number of valid pixels	—	2.5 × 10^6^	4.2 × 10^6^	—
*P*_*g*_	Number of green pixels	—	0	4.2 × 10^6^	—
*P*_*s*_	Number of pixels per CLS spot	—	0	4.2 × 10^6^	—
*N*_*s*_	Number of spots	—	0	2000	—
*A*	Pixel size at the ground level	mm^2^	0.13	0.13	—
GF	Green fraction	—	0	1	$\text{GF}=\frac{P_{g}}{P_{v}}$
SD	Spot density	cm^−2^	0	0.4	$\text{SD}=\frac{N_{s}}{P_{v}\cdot A\cdot 10^{-2}}$
SS	Average spot size	mm	2	5	$\text{SS}=\frac{4}{\pi Ns}\underset{\text{spots}}{\sum }\sqrt{P_{s}\cdot A}$

**Table 3:** Estimation results obtained using every possible combination of image-derived features (GF, GFn, SD, and SS for Phenomobile; GF and GFn for UAV) as inputs to the neural network. RMSE are estimated using twofold cross-validation (2016 for training and 2017 for validation, and reciprocally) and 20 replicates. For each vector, results are sorted from minimum RMSE to maximum RMSE.

Vector	Variables extracted from images				RMSE
	GF	GFn	SD	SS	
Phenomobile	—	✓	✓	✓	0.91
	—	✓	✓	—	0.98
	✓	✓	✓	✓	0.99
	✓	✓	✓	—	1.05
	—	—	✓	✓	1.08
	—	—	✓	—	1.09
	✓	—	✓	—	1.15
	✓	—	✓	✓	1.18
	—	✓	—	—	1.19
	✓	✓	—	—	1.33
	—	✓	—	✓	1.40
	✓	✓	—	✓	1.43
	✓	—	—	—	2.30
	✓	—	—	✓	2.37
	—	—	—	✓	2.96

UAV	—	✓	—	—	1.23
	✓	✓	—	—	1.46
	✓	—	—	—	2.35

**Table 4:** Comparison between the three scoring methods used in this study based on seven criteria.

Criterion	Visual scoring	Phenomobile RGB imagery	UAV multispectral imagery
Accuracy (scores)	+++	++++	++
Accuracy (ADPC)	+++	++++	+++
Specificity	++++	+++	+
Representativeness	++++	+++	++++
Repeatability	+++	++++	+++
Efficiency	+	++	++++
Affordability	++++	+	+++

## Data Availability

The data used for this paper are freely available upon request.

## Supplementary Materials

Supplementary Materials Table S1: confusion matrix obtained for the SVM classification of green and nongreen pixels in Phenomobile RGB images. Figure S1: typical temporal courses observed for GF (Phenomobile estimation for column 1 and UAV estimation for column 4), SD (column 2), and SS (column 3) and for 2016 (row 1) and 2017 (row 2). Orange squares correspond to the measured values, and green circles correspond to the values interpolated at the visual scoring dates. Time is expressed in growing degree days (GDD) after disease inoculation. Figure S2: diagram of the neural network used to estimate CLS scores from the *n* input(s) *X*_1_, ⋯, *X*_*n*_ (to be chosen among GF, GFn, SD, and SS). Figure S3: distributions of visual scores in 2016 (left) and 2017 (right).
