# Supplementary material for: Scoring Cercospora Leaf Spot on Sugar Beet: Comparison of UGV and UAV Phenotyping Systems
Source: Plant Phenomics. 2020 Aug 5;2020:9452123. doi: 10.34133/2020/9452123 (PMC7706347; doi:10.34133/2020/9452123)
Supplement: Supplementary Materials — Table S1: confusion matrix obtained for the SVM classification of green and nongreen pixels in Phenomobile RGB images. Figure S1: typical temporal courses observed for GF (Phenomobile estimation for column 1 and UAV estimation for column 4), SD (column 2), and SS (column 3) and for 2016 (row 1) and 2017 (row 2). Orange squares correspond to the measured values, and green circles correspond to the values interpolated at the visual scoring dates. Time is expressed in growing degree days (GDD) after disease inoculation. Figure S2: diagram of the neural network used to estimate CLS scores from the n input(s) X1, ⋯, Xn (to be chosen among GF, GFn, SD, and SS). Figure S3: distributions of visual scores in 2016 (left) and 2017 (right). [file 9452123.f1.docx]

**Table S1: Confusion matrix obtained for the SVM classification of green and non-green pixels in Phenomobile RGB images.**

|  | | Actual classes | | User’s accuracy |
| --- | --- | --- | --- | --- |
|  |  | Non-green | Green |  |
| Predicted classes | Non-green | 451 | 8 | 0.98 |
|  | Green | 7 | 433 | 0.98 |
| Producer’s accuracy | | 0.98 | 0.98 | Overall accuracy = 0.98 |


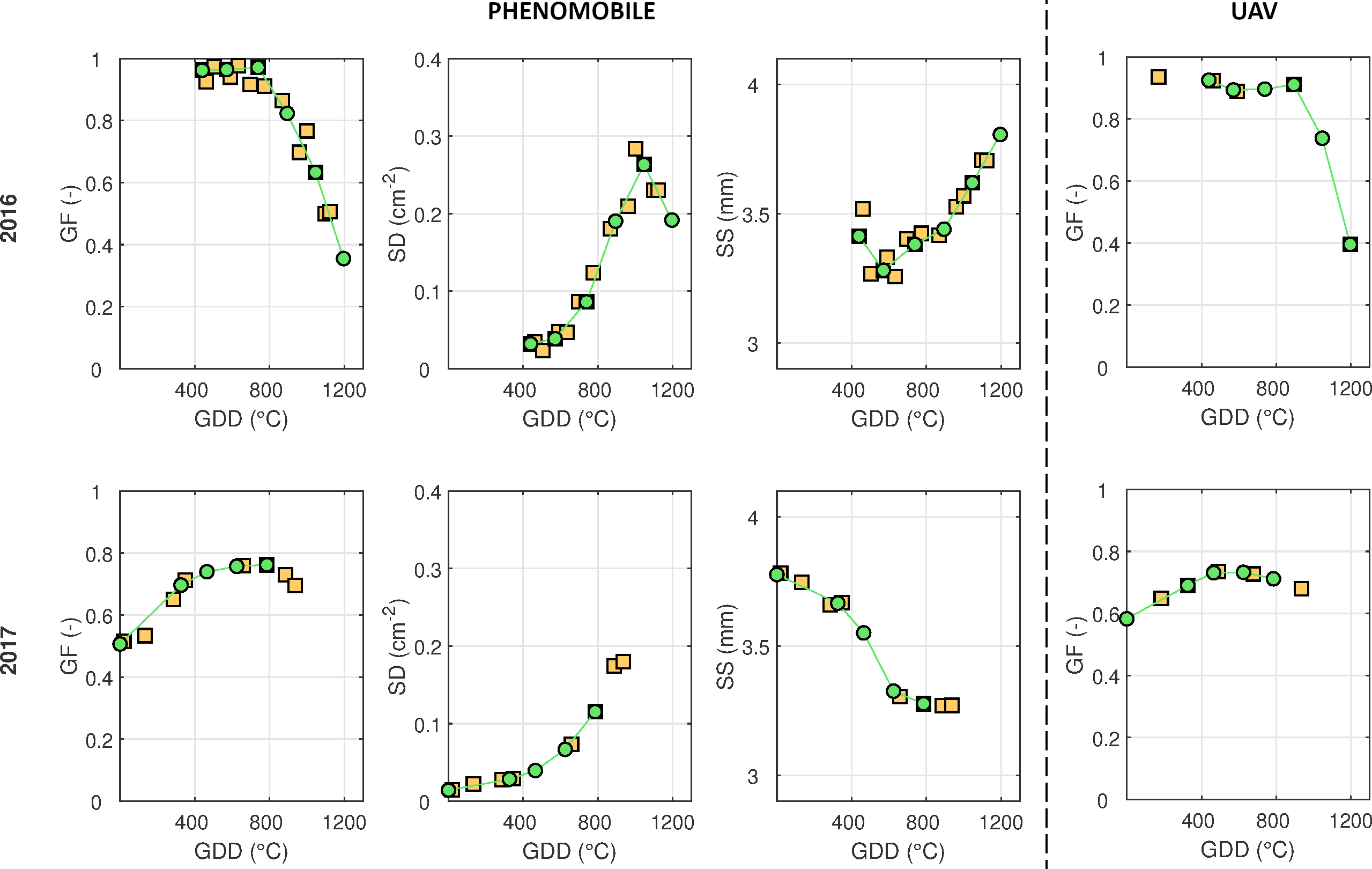


**Figure S1: Typical temporal courses observed for GF (Phenomobile estimation for column 1 and UAV estimation for column 4), SD (column 2) and SS (column 3), and for 2016 (row 1) and 2017 (row 2).** Orange squares correspond to the measured values, and green circles correspond to the values interpolated at the visual scoring dates. Time is expressed in growing degree days (GDD) after disease inoculation.


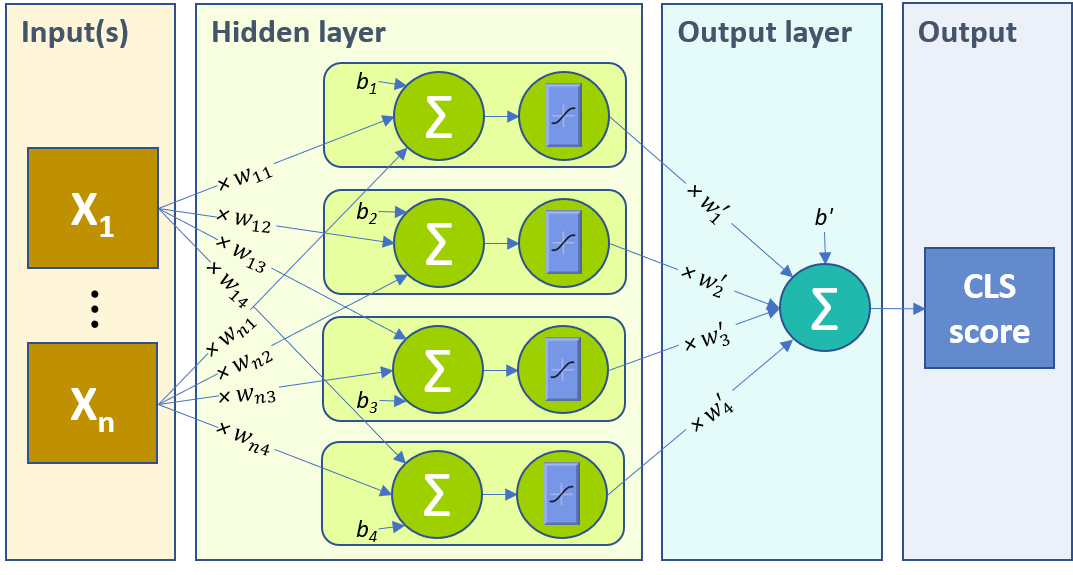


**Figure S2: Diagram of the neural network used to estimate CLS scores from the n input(s) X_1_, …, X_n_.** **(to be chosen among GF, GFn, SD and SS).**


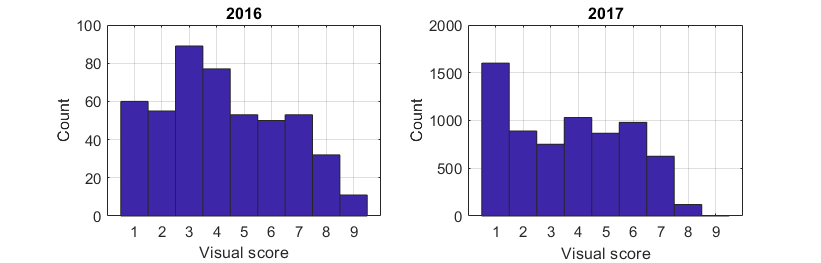


**Figure S3: Distributions of visual scores in 2016 (left) and 2017 (right).**
